# Proteomic and functional profiling of platelet-derived extracellular vesicles released under physiological or tumor-associated conditions

**DOI:** 10.1038/s41420-022-01263-3

**Published:** 2022-11-26

**Authors:** Mauro Vismara, Marcello Manfredi, Marta Zarà, Silvia Maria Grazia Trivigno, Luca Galgano, Silvia Stella Barbieri, Ilaria Canobbio, Mauro Torti, Gianni Francesco Guidetti

**Affiliations:** 1 Department of Biology and Biotechnology “Lazzaro Spallanzani”, University of Pavia, 27100 Pavia, Italy; 2 Department of Translational Medicine, University of Piemonte Orientale, 28100 Novara, Italy; 3 Unit of Brain-Heart Axis: Cellular and Molecular Mechanism, Centro Cardiologico Monzino IRCCS, 20138 Milan, Italy; 4 University School for Advanced Studies IUSS, Pavia, Italy

**Keywords:** Protein-protein interaction networks, Signal transduction, Cancer microenvironment

## Abstract

During hemostasis, thrombosis, and inflammation, activated blood platelets release extracellular vesicles (PEVs) that represent biological mediators of physiological and pathological processes. We have recently demonstrated that the activation of platelets by breast cancer cells is accompanied by a massive release of PEVs, evidence that matches with the observation that breast cancer patients display increased levels of circulating PEVs. A core concept in PEVs biology is that their nature, composition and biological function are strongly influenced by the conditions that induced their release. In this study we have performed a comparative characterization of PEVs released by platelets upon activation with thrombin, a potent thrombotic stimulus, and upon exposure to the breast cancer cell line MDA-MB-231. By nanoparticle tracking analysis and tandem mass spectrometry we have characterized the two populations of PEVs, showing that the thrombotic and tumoral stimuli produced vesicles that largely differ in protein composition. The bioinformatic analysis of the proteomic data led to the identification of signaling pathways that can be differently affected by the two PEVs population in target cells. Specifically, we have demonstrated that both thrombin- and cancer-cell-induced PEVs reduce the migration and potentiate Ca^2+^-induced apoptosis of Jurkat cells, but only thrombin-derived PEVs also potentiate cell necrosis. Our results demonstrate that stimulation of platelets by thrombotic or tumoral stimuli induces the release of PEVs with different protein composition that, in turn, may elicit selective biological responses in target cells.

## Introduction

Platelets are anucleated blood cells primarily involved in hemostasis and thrombosis and are activated by adhesion molecules and soluble agonists. Platelet activation stimulates a complex array of signaling pathways, leading to platelet aggregation and blood clot formation [1]. These processes are accompanied by the release of extracellular vesicles (EVs), commonly defined as platelet-derived extracellular vesicles (PEVs), that are mediators of intercellular communication [2]. By delivering biologically active molecules to target cells, PEVs regulate several processes and are involved in the onset and progression of different pathological conditions [3]. In response to various stimuli, blood platelets release in the bloodstream a heterogenous population of PEVs mainly composed by plasma membrane-derived PEVs and exosomes [2]. PEVs generated by budding of the plasma membrane have a diameter ranging from 100 nm to 1 μm and are commonly referred to also as platelet-derived microparticles (PMPs), platelet-derived microvesicles (PMVs), or medium-large PEVs. These medium/large PEVs (henceforth defined PEVs) incorporate proteins, lipids, and nucleic acids, and their specific composition is known to depend on the environmental conditions and the stimuli that induce their release [2].

A growing body of evidence suggests that PEVs are also important regulators of cancer progression and modulate key steps of the metastatic cascade [4, 5]. Specifically, PEVs display the remarkable ability to bind tumor cells and to regulate their survival and intrinsic aggressiveness [6–12]. In previous studies, we have characterized the mechanism of platelet activation induced by breast cancer cells [13], and we have demonstrated that breast cancer cells can also induce the release of PEVs [7]. More recently, also colon cancer cells were shown to stimulate the generation of PEVs [14], suggesting that this ability may be a common feature of cancer cells. To date, nothing is known about the composition and function of PEVs released by platelets upon exposure to cancer cells, and whether they differ in any way from PEVs released in response to classical hemostatic/thrombotic stimuli.

In this study, PEVs released by platelets upon exposure to the breast adenocarcinoma cell line MDA-MB-231 or upon stimulation with thrombin were isolated and characterized through a proteomic approach to unravel specific differences in the cargo proteins associated with possible distinctive functional properties.

## Results

### Analysis of PEVs size distribution

In this study, we characterized and compared two different populations of PEVs, named as follows:

1. PEVs-THR, released by platelets in response to the physiological stimulus thrombin;
2. PEVs-BCC, released by platelets in response to the incubation with the triple negative, highly aggressive breast cancer cell line MDA-MB-231.

**Fig. 1:**
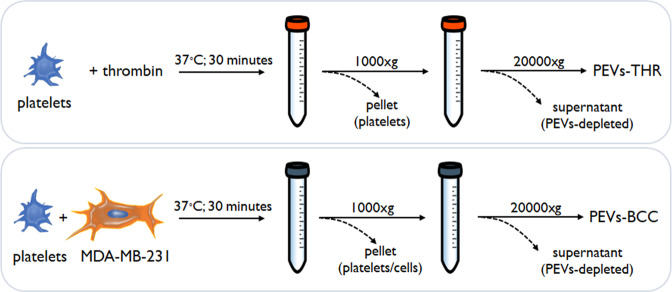
Graphical summary of the PEVs isolation procedure. Each sample of PEVs was obtained by the stimulation of platelets (5 ml at the concentration of 3 × 10^8^/ml) with 0.1 U/ml thrombin or MDA-MB-231 cells (5 × 10^4^ cell/ml).

The procedure for PEVs isolation (Fig. 1) was based on the strategy adopted in our previous investigations [6, 7, 12], and is detailed in the materials and methods section. The two populations of PEVs have been previously analyzed for their ability to stimulate the intrinsic aggressiveness of MDA-MB-231 cells [6, 7, 12] but they have not been characterized for their distinctive molecular signature.

The NTA analysis revealed that platelet stimulation with thrombin or with MDA-MB-231 cells resulted in the release of 6.20 ± 1.54 × 10^9^ and 7.94 ± 5.0 × 10^9^ particles per sample, respectively. PEVs purified from each sample contained an average of about 70 µg of proteins (PEVs-THR: 72.6 ± 4.4 µg; PEVs-BCC: 67.55 ± 5.93 µg).

**Fig. 2:**
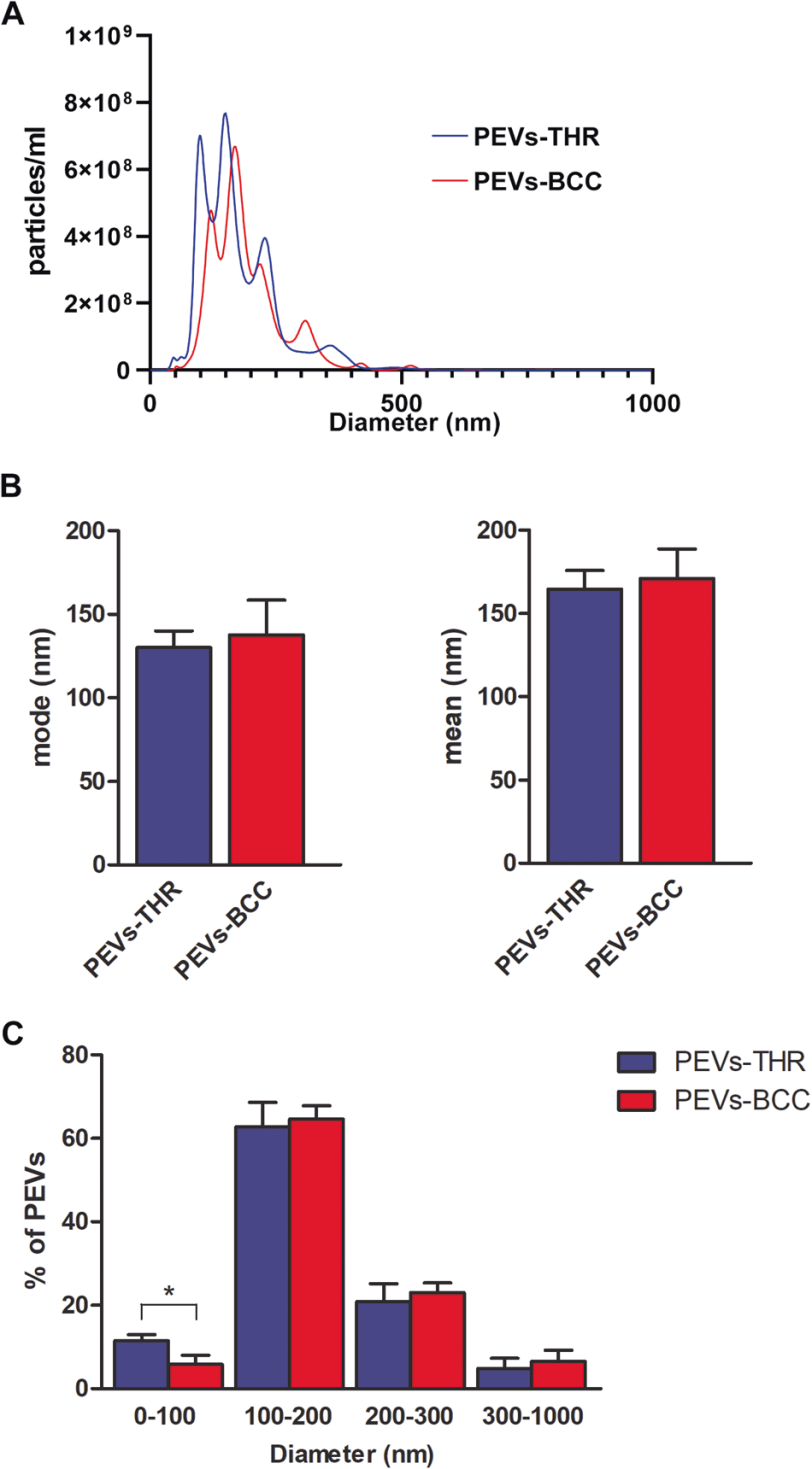
Analysis of size distribution of PEVs. **A** Representative size distribution of PEVs-THR and PEVs-BCC obtained by NanoSight analysis. **B** Quantification of the analysis of PEVs size expressed as mode (i) and mean (ii) diameter. Results are reported as the mean ± SD of three independent experiments. **C** Size distribution expressed as the percentages of particles detected in the different dimension ranges (0–100, 100–200, 200–300, 300–1000 nm). The results are reported as the mean ± SD of three experiments. Statistical significance of the differences was calculated between PEVs-THR and PEVs-BCC within the same size range. *p < 0.05.

The measurement of the size distribution of PEVs showed that both preparations contained heterogenous populations of vesicles with size ranging from 50 to 500 nm (Fig. 2A). Data analysis revealed that both the mode and mean size of PEVs-THR and PEVs-BCC were similar (Fig. 2B). In both PEVs preparations most of the particles (about 60%) were in the 100–200 nm diameter range, whereas about 20% of PEVs had a size in the 200–300 nm range (Fig. 2C). The percentage of vesicles with a diameter higher than 300 nm was below 10% in both samples. Interestingly, a statistically significant difference between the two preparations of PEVs was observed for the smallest vesicles, with a diameter below 100 nm: the percentage of PEVs-THR falling in this subpopulation was clearly higher than that of PEVs-BCC (11.5% ± 1.49 vs 5.80% ± 2.25).

### Comparative proteomic profiling of PEVs-THR and PEVs-BCC

**Fig. 3:**
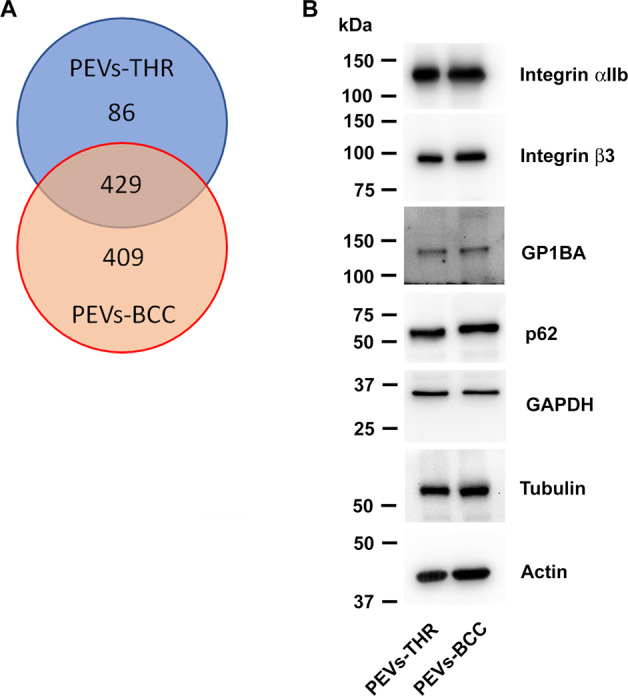
Proteomic profiling of PEVs. **A** Venn diagram reporting the number of proteins identified in the PEVs-THR and PEVs-BCC samples by LC-MS. **B** Representative immunoblots of selected proteins expressed in PEVs: integrin αIIb, integrin β3, GPIbα (GP1BA), p62, GAPDH, tubulin and actin, as indicated.

The protein content of the two populations of PEVs was analyzed upon in-solution digestion by liquid chromatography-tandem mass spectrometry. The list of proteins identified in the two PEVs samples analyzed is reported as supplementary material (Table S1). As shown in the Venn diagram reported in Fig. 3A, a total of 924 proteins were identified through this approach, of which 429 proteins found in both samples, 86 proteins exclusively present in the PEVs-THR, and 409 proteins detected only in the PEVs-BCC. It appears therefore that PEVs-BCC are more heterogeneous in protein composition than PEVs-THR. Platelet-derived proteins, such as integrin αIIb (ITA2B), integrin β3 (ITB3), and GPIbα (GP1BA), as well as the cytoskeletal proteins actin (ACTB) and tubulin (TBA1), were found in both PEVs preparations and their presence was confirmed by immunoblotting analysis. Also typical EVs markers such as the enzyme glyceraldehyde-3-phosphate dehydrogenase (GAPDH) and the autophagosome component p62 [15, 16], were found in both PEV-THR and PEV-BCC as also confirmed by immunoblotting (Fig. 3B). The presence of several proteins selectively found in one of the two PEVs preparations (marked in blue in Table S1) suggests that the mechanism of cargo selection for PEVs-THR and PEVs-BBC may be significantly different and may underlie radically different functional abilities.

As a comparison, vesicles directly released by cultured MDA-MB-231 cells (henceforth named EVs-MDA-MB-231) were also subjected to proteomic profiling. To this purpose, MDA-MB-231 cells were incubated under the same experimental conditions used to generate PEVs-BCC, but in the absence of platelets. A limited amount of EVs was recovered in the supernatant of these samples (0.47 ± 0.21 µg). LC-MS analysis identified 566 proteins in EVs-MDA-MB-231. Only a relatively small fraction (135 proteins out of 566) was shared by EVs-MDA-MB-231 and PEVs-BCC, while 166 proteins were found in all the EVs populations. As expected, very few proteins, specifically 4, were identified in both EVs-MDA-MB-231 and PEVs-THR (Supplementary Fig. 1).

### Quantitative MS analysis of PEVs-THR and PEVs-BCC

**Fig. 4:**
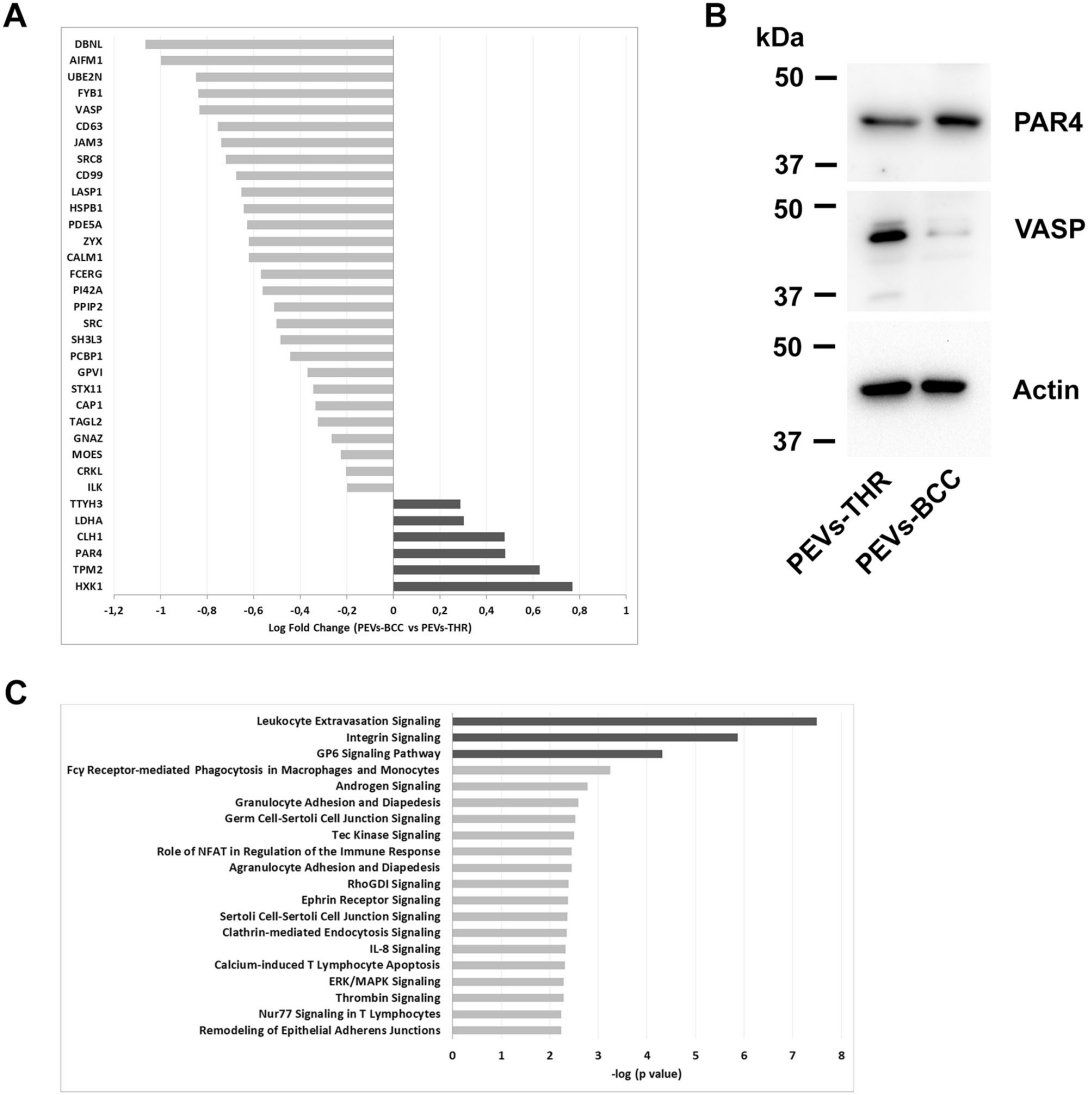
Quantitative proteomic and gene ontology enrichment analysis. **A** Protein quantification in PEVs populations by LC-MS. The Log Fold Change indicates the difference of single protein expression in PEVs-BCC vs PEVs-THR. The dark gray bars indicate proteins more represented in PEVs-BCC, conversely light gray bars shown proteins more enriched in PEVs-THR. **B** Immunoblotting analysis of PAR4 and VASP in PEVs-THR and PEVs-BCC. Actin staining was performed as control for equal loading. **C** Gene ontology enrichment analysis of the PEVs proteins involved in the regulation of specific biological processes. The 20 pathways that, according to the bioinformatic analyses, displayed the most significant protein enrichment are reported. The dark gray bars indicate pathways that were predicted to be inhibited by PEVs-BCC compared to PEVs-THR, whereas light gray bars indicate processes that are likely to be modulated although no prediction was possible.

The ability of PEVs to regulate specific processes in target cells has been widely documented [6–9, 12, 17–19]. It is reasonable to hypothesize that the capacity of PEVs to control biological responses may depend on the delivery of proteins involved in the regulation of specific pathways. Since PEVs-THR and PEVs-BCC are remarkably different in protein composition, we further investigated their protein cargo by quantitative proteomic analysis. While qualitative analysis can provide a list of proteins identified in PEVs samples, label-free quantitative proteomics yields information about the functional differences between two biological samples. This approach allowed the comparison of the levels of proteins expressed in PEVs-THR and PEVs-BCC. A total of 34 proteins were found to be differentially expressed between the two PEVs samples (fold change > 1.3 and *p*-value < 0.05) (Fig. 4A). Light gray bars indicate proteins that are expressed at higher levels in PEVs-THR rather than PEVs-BCC, conversely dark gray bars indicate proteins that are more abundant in PEVs-BCC. The results from quantitative MS analysis were validated by immunoblotting, focusing on the proteins VASP and PAR4, which are detectable in both samples, but expressed at higher levels in PEVs-THR and PEVs-BCC, respectively (Fig. 4B).

Bioinformatic analysis of the expression profiles allowed us to associate specific groups of differently expressed proteins to predict functional pathways. Proteins involved in the regulation of leukocyte extravasation, integrin, and GPVI signaling, were found to be more abundant in PEVs-THR than in PEVs-BCC. Accordingly, these pathways were also predicted to be negatively regulated in PEVs-BCC versus PEV-THR (Fig. 4C, dark gray bars). At the same time, other pathways (light gray bars in Fig. 4C), such as ERK/MAPK signaling and Calcium-induced T Lymphocyte Apoptosis, were identified as significantly altered by PEVs, but here a prediction of regulation, inhibition or activation, was not possible based on the proteomic quantitative profile (Fig. 4C). A complete list of all the predicted pathways, potentially associated with the proteins quantified in PEVs, is reported in Supplementary Table S2.

### PEVs in the regulation of leukocyte functions

In search of novel functional effects of PEVs, we chose to focus on their possible ability to regulate leukocyte function. In particular, the effects of PEVs-THR and PEVs-BCC on the regulation of leukocyte extravasation and calcium-induced T-cell apoptosis were investigated. As experimental cellular model, the immortalized human T-cell line Jurkat was selected, since these cells have been previously used to investigate leukocyte migration [20, 21] and Ca^2+^-induced apoptosis [22, 23].

**Fig. 5:**
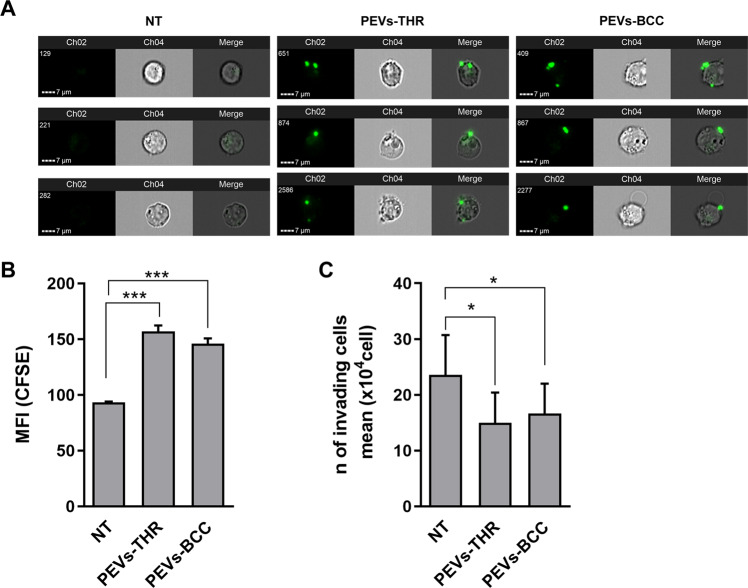
Interaction of PEVs with Jurkat cells and analysis of cell invasion. **A** Representative images of CFSE-loaded PEVs interacting with Jurkat cells. For Jurkat cells treated with PEVs-THR or PEVs-BCC and untreated cells (NT), three representative examples including green-fluorescence images of Channel 2 (Ch2), brightfield images of Channel 4 (Ch4) and Merge of Ch2 and Ch4 are shown. **B** Analysis of PEVs-Jurkat cells interaction by flow cytometry. Jurkat cells were co-cultured for 24 h with PEVs obtained from CFSE-labeled platelets upon stimulation with thrombin or MDA-MB-231 cells. The green-fluorescent signal of PEVs associated to Jurkat cells was quantified as Median Fluorescence Intensity (MFI). The plot reports the mean ± SD of three independent experiments. ***p < 0.0001 (sample vs NT). **C** Jurkat cells migration evaluated by transwell assay. Cells that moved in the bottom chamber were collected and counted using a hemocytometer. The data are expressed as number of migrated cells and the graph shown the mean ± SD of four independent experiments with *p < 0.05.

By imaging flow cytometry experiments, we found that Jurkat cells efficiently interacted with PEVs upon 16 h incubation (Fig. 5A). Quantitative analysis revealed that PEVs-THR and PEVs-BCC displayed a comparable ability to bind to Jurkat cells (Fig. 5B).

To study leukocyte extravasation, a transwell assay with matrigel-coated membranes was adopted. This approach revealed that incubation with PEVs significantly reduced the chemotactic invasion of Jurkat cells through the extracellular matrix. Despite the different enrichment in proteins regulating the extravasation pathway, PEVs-THR and PEVs-BCC displayed a very similar effect on Jurkat cell migration (Fig. 5C). Conversely, EVs released by cancer cells in the absence of platelets (EVs-MDA-MB-231) did not influence Jurkat cell migration (Supplementary Fig. 2).

**Fig. 6:**
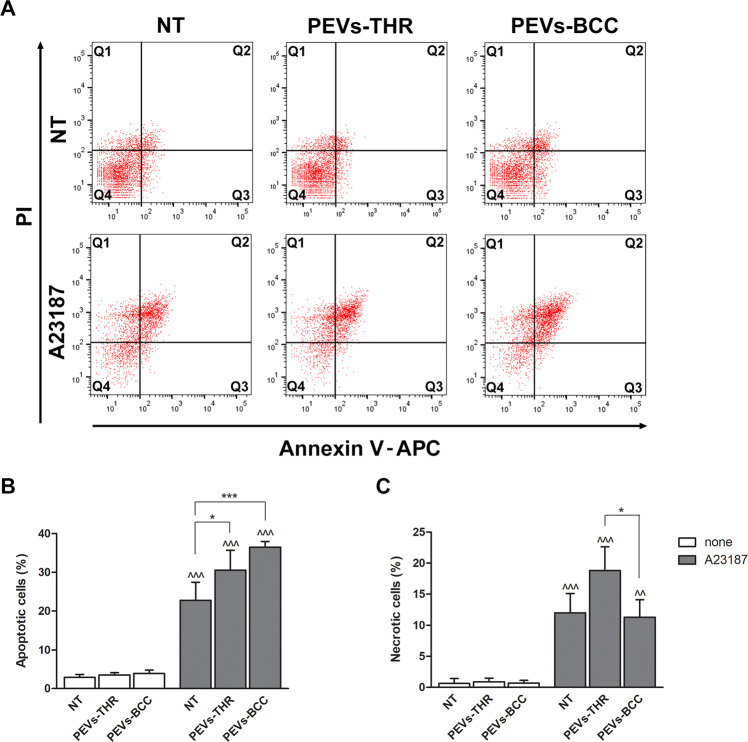
Effect of PEVs on calcium-induced apoptosis in Jurkat cell. **A** Representative dot plots of Jurkat cell apoptosis analyzed by flow cytometry. The signal associated to propidium iodide (PI) and Annexin V-APC are reported on the y- and *x*-axis, respectively. **B** Quantification of apoptosis as the percentage of cells in the Q2 (late apoptosis) and Q3 (early apoptosis) quadrants. The data are representative of the mean ± SD of four independent experiments. The results of the statistical analysis of the differences between samples treated with A23187 (gray bars) and respective controls (white bars) are indicated by ^^^ (*p* < 0.0001). The statistical significance of the differences between PEVs-treated samples and respective controls is indicated with the asterisks (*p < 0.05; ***p < 0.0001). **C** Quantification of necrotic cells as the percentage of cells in the Q1 quadrant. The data are representative of the mean ± SD of three independent experiments with *p < 0.05. The statistical significance between samples treated with A23187 (gray bars) and respective controls (white bars) are indicated by as ^ (*p* < 0.05) and ^^ (*p* < 0.001).

We next investigated whether the interaction with PEVs could influence Ca^2+^-induced apoptosis in Jurkat T cells, as predictable from the informatic analysis of PEVs cargo. As reported in Fig. 6, the incubation of Jurkat cells with PEVs-THR or PEVs-BCC alone did not cause any significant increase of the percentage of apoptotic cells. As expected, stimulation with the Ca^2+^ ionophore A23187 induced a remarkable apoptosis in Jurkat cells. The percentage of apoptotic cells significantly increased when stimulation of Jurkat cells with the Ca^2+^ ionophore A23187 was performed in the presence of PEVs (Fig. 6B). Although PEVs-BCC appeared to display a more potent stimulation of apoptosis than PEV-THR, the statistical analysis revealed no significant differences between the two samples. The treatment with the Ca^2+^ ionophore A23187 caused necrosis in about 10% of the Jurkat cells. Interestingly, Ca^2+^-induced T-cell necrosis was significantly potentiated in the presence of PEVs-THR but was not affected by PEVs-BCC (Fig. 6C). Therefore, both PEVs-THR and PEVs-BCC were able to stimulate Ca^2+^-induced apoptosis of Jurkat cells, but only PEVs-THR also potentiated cell necrosis.

## Discussion

In the present study, we have combined proteomic and cell biology approaches to provide a characterization of EVs released by platelets upon incubation with the triple negative cell line MDA-MB-231, in comparison with EVs released upon a thrombotic stimulus. Our results demonstrate a remarkable heterogeneity of the protein cargo in the two different types of PEVs, that is partially mirrored by differences in the ability to modulate leukocyte function.

PEVs are important regulators of cancer progression: by transferring bioactive molecules from platelets to cancer cells, PEVs modulate specific features of neoplastic cells and tumor microenvironment [4].

The majority of previous studies aimed to understand the functional effects of PEVs on target cells in the context of cancer, focused their attention on medium/large PEVs released in response to stimulation with common platelet agonists, such as thrombin and collagen [6, 8, 9, 12, 17, 18]. We have previously demonstrated that cancer cells themselves can activate platelets and induce a massive release of PEVs [7]. This observation matches the notion that the levels of circulating medium/large PEVs are elevated in cancer patients [24–27], and indicates that the direct interaction between platelets and circulating cancer cells may play an active role in the release of PEVs. Here we addressed the question whether these PEVs released upon activation by cancer cells are different from PEVs released upon platelet stimulation with physiological agonists in terms of protein cargo and functional properties.

A major conclusion of our study is that PEVs composition is significantly different, depending on the stimulus that induces their release. The proteomic profiling demonstrated that some proteins, including cytoskeletal proteins and typical platelet-derived membrane receptors, are common between PEVs-THR and PEVs-BCC, but several others were found uniquely in one type of PEVs. PEVs-BCC are characterized by a more complex composition and more than 400 proteins detected in these vesicles, were not found in PEVs-THR. By a comparative analysis with previous data sets of platelet proteomic analyses [28], we found that the majority (about 75%) of the proteins exclusively detected in PEVs-BCC were expressed in platelets. This observation supports the concept that tumor cells are able to promote a specific sorting of selected proteins into released PEVs. About 25% of proteins found in PEVs-BCC were not previously detected in platelets, suggesting that some tumor cell proteins could be incorporated into the released PEVs, which may actually have a hybrid identity. It may be considered that the supernatant of platelets co-cultured with MDA-MB-231 cells could contain also vesicles originating from cancer cells, rather than from platelets. Fully addressing these aspects is a demanding task and goes beyond the goal of our current interest. However, we have previously demonstrated that platelets do not stimulate the release of EVs from co-cultured BCCs and that EVs recovered in the supernatant are exclusively of platelet origin [7]. Furthermore, proteomic analysis of vesicles spontaneously released by MDA-MB-231 cells (EVs-MDA-MB-231) revealed that relatively few proteins were in common with PEVs-BCC. Our analysis also identified proteins in PEVs-BCC, such as integrin alpha3 subunit and tissue factor, that are expressed in cancer cells but not in platelets [28], and that were not detected in EVs-MDA-MB-231. Based on these observations, it is reasonable to conclude that while cancer cells activate platelets inducing the release of PEVs, a feedback regulation exerted by platelets on MDA-MB-231 cells can promote the loading of some cancer-derived proteins into the released vesicles. Therefore, the proteomic composition of PEVs-BCC likely derives from a complex functional interplay between platelets and cancer cells, rather than being a simple mixture of two populations of distinct vesicles independently released by the two cell types.

Quantitative protein profiling coupled with bioinformatic analysis allowed us to identify functional pathways that could be potentially regulated by PEVs in target cells. Some of these signaling pathways, including MAPK/ERK pathway, have already been investigated in previous studies in target cancer cells [6, 7].

In addition to interacting directly with cancer cells, platelets are known to regulate leukocytes [4, 29], but the possible contribution of PEVs in this frame is poorly known. Here we focused our attention on two still unexplored pathways related to the regulation of leukocyte function, namely leukocyte extravasation and the Ca^2+^-induced apoptosis, using Jurkat cells as experimental cellular model.

Based on the presence of several proteins involved in leukocyte motility among the protein cargo of both types of PEVs we predicted that migration of Jurkat cells could be regulated by PEVs, as already observed for other cell types [6, 9]. Our results indicated that Jurkat cells bind equally well PEVs-THR and PEV-BCC and that this interaction was associated to a reduced cell invasiveness. Moreover, despite the prediction by the bioinformatic analysis of potentially different effects of PEVs-THR and PEVs-BCC, they were equally efficient in reducing Jurkat cell mobility. The mechanisms supporting this effect deserve further investigations, although it may be hypothesized that the negative regulation of Jurkat cell migration is due to PEVs bioactive components different than proteins that remain to be identified and that are equally expressed in both types. However, the observation that PEVs alone did not cause significant apoptosis or necrosis of Jurkat cells (Fig. 6), ruled out the possibility that the reduced migration was consequent to reduced cell viability.

We also found that the physical interaction with PEVs sensitized Jurkat cells to Ca^2+^-induced apoptosis. To the best of our knowledge, this observation represents the first evidence that PEVs can regulate T-cell apoptosis and may have important implications in cancer progression. It can be hypothesized that, by activating platelets and inducing the release of PEVs, cancer cells could potentiate T-cell apoptosis as an additional mechanism of immune evasion. Once again, PEVs-THR and PEVs-BCC were similarly efficient in sensitizing Jurkat cells to Ca^2+^-induced apoptosis, although PEVs-BCC displayed a slightly stronger effect. However, only PEVs-THR but not PEVs-BCC potentiated Ca^2+^-induced cell necrosis. The bioinformatic analysis failed to identify the molecular signature that supports such a different behavior and no obvious explanation emerged from the critical analysis of the differential protein enrichment between the two populations of PEVs. We performed a preliminary analysis of the signaling pathways possibly regulated by PEVs in this context. Interestingly, co-culture of Jurkat cells with both populations of PEVs in the absence of Ca^2+-^ ionophore was associated to the increase of Akt phosphorylation, but only PEVs-BCC caused a modest reduction of p38MAPK and ERK (p42/44 MAPK) phosphorylation. Upon treatment with A23187, the phosphorylation of all these proteins was strongly reduced independently of the presence of PEVs, likely as a consequence of major cell disruption associated with apoptosis (Supplementary Fig. 3). These observations confirm that PEVs are able to perturbate Jurkat cell homeostasis, although the precise mechanism linking PEVs to Ca^2+^-induced apoptosis certainly deserves further investigation. Nonetheless, it was noted that PEVs-THR expressed significantly higher amounts of apoptosis-inducing factor mitochondria-associated 1 (AIFM1), which was previously associated to necrotic-like mechanisms of cell death [30] and may be involved in the potentiation of necrosis observed in our experiments. Moreover, it cannot be excluded that other classes of bioactive molecules delivered by the two PEVs populations could be responsible for the diverse effect on cell necrosis.

## Conclusions

Our results indicate that tumor-cell-induced platelet activation leads to the generation of PEVs that are similar in quantity and size to those generated in response to a thrombotic stimulus, but that are characterized by a different protein cargo. We have identified two previously unknown effects of PEVs on leukocytes, demonstrating that, despite the different protein enrichment, PEVs-THR and PEVs-BCC inhibit Jurkat cell migration and potentiate Ca^2+^-induced apoptosis. Moreover, only PEVs-THR but not PEVs-BCC stimulate necrosis of Jurkat cells.

Altogether, these data represent a useful platform for further studies in search of still unknown functional effects of PEVs and open the way to new models in the platelet-cancer interplay.

## Materials and methods

### Materials

DMEM, RPMI, D-PBS, FBS, L-glutamine, penicillin/streptomycin, and BCA protein Assay Kit were from EuroClone. Trypan Blue, thrombin, trypsin, DTT, iodoacetamide, Propidium Iodide (PI), Matrigel, CFSE, and A23187 were purchased from Sigma–Aldrich. Annexin V-APC was from BD Biosciences. The antibodies for integrin αIIb (B-10), integrin β3 (C-20), tubulin (DM1A), and glyceraldehyde-3-phosphate dehydrogenase (G-9) were from Santa Cruz Biotechnology. The antibody against PAR4 and VASP were from ABclonal. The antibody for GPIbα (EPR6995) and p62 (2C11) were purchased from Abcam, whereas anti Actin (AC-15) was from Sigma–Aldrich. The antibodies for phospho-Akt (Ser473), phospho-p38 MAPK (Thr180/Tyr182), and phospho-p44/42 MAPK (ERK1/2) (Thr202/Tyr204) were from Cell Signaling Technology.

### Cell culture

MDA-MB-231 cells were cultured as previously described [6]. Jurkat cells were maintained in culture at a concentration between 1 × 10^5^ and 1 × 10^6^ viable cells/ml in RPMI supplemented with 10% FBS, 2 mM L-glutamine, 100U/ml penicillin, and 100 μg/ml streptomycin. The day of the experiment, cells were harvested, centrifuged at 120 × *g* for 10 min, and resuspended in the culture medium at the desired concentration.

### PEVs isolation

Human blood platelets were purified from buffy coat bags as previously described [31]. Washed platelets were resuspended at the concentration of 3 × 10^8^/ml in HEPES buffer supplemented with 1 mM CaCl_2_, 0.5 mM MgCl_2_, and 5.5 mM glucose. To induce the release of PEVs, samples containing 5 ml of washed platelets were stimulated for 30 min at 37 °C under constant stirring with 0.1 U/ml of thrombin or with MDA-MB-231 cells (5 × 10^4^ cells/ml) in the presence of 0.05% v/v of autologous platelet poor plasma. As control, MDA-MB-231 cells were incubated under the same experimental conditions in the absence of platelets, to produce EVs of cancer cell origin.

Platelets and cells were pelleted by low-speed centrifugation (750 × *g*, 20 min) and the supernatant was then centrifuged at 20,000 × *g* for 90 min at 10 °C to collect PEVs. PEVs were resuspended in 25 mM Tris/HCl, 0.1% SDS, pH 7.4 for proteomic analysis and in HEPES buffer for all the other experiments, and the protein content was determined by BCA protein assay kit.

In selected experiments, platelets were labeled with 3 μg/ml of CFSE for 10 min before stimulation, to obtain fluorescently labeled PEVs for the analysis of their interaction with Jurkat cells.

### Nanoparticle tracking analysis

The concentration and dimension of purified PEVs were assessed by Nanoparticle Tracking Analysis (NTA) using NanoSight NS300 (Malvern Panalytical). The optimal dilution for the NTA analysis was determined and, for each sample, five videos of 60 sec were recorded and analyzed using NTA software (version 3.4; NanoSight Ltd.).

### Immunoblotting

The analysis of proteins of PEVs was performed by immunoblotting, as described previously [32]. Membrane staining was performed using specific primary antibodies diluted 1:1000 in TBS (20 mM Tris, 500 mM NaCl, pH 7.5) containing 5% BSA and 0.1% Tween-20 in combination with the appropriate HRP-conjugated secondary antibodies (1:2000 in PBS plus 0.1% Tween-20). Acquisition of membrane images was performed by ChemiDoc XRS Imaging System (Bio-Rad).

### Proteomic analysis

The proteomic analyses were performed as previously described [33, 34], and the detailed protocol is available as supplementary file.

### Analysis of PEVs interaction with Jurkat cells

Jurkat cells (10^6^ cells/well) were seeded in a 24-well plate and incubated for 16 h with 30 μg/ml of PEVs obtained from stimulated CFSE-labeled platelets. Negative control samples were cultured under the same conditions in the absence of PEVs. Cells were subsequently collected by centrifugation at 700 × *g* for 7 min and washed once with PBS. Cells were then recovered by centrifugation, resuspended in PBS and eventually fixed with 0.5% of PFA for 15 min at room temperature in the dark. The interaction between PEVs and Jurkat cells was analyzed by flow cytometry (BD FACSLyric, BD Biosciences) and by imaging flow cytometry (Amnis ImageStream X Mark II, Merck Millipore).

### Cell migration assay

The effect of PEVs on migration of Jurkat cells was evaluated using Falcon cell culture inserts (8 μm pore size) coated with 15 µg of Matrigel positioned in a 24-well plate [35]. Cells were resuspended in RPMI containing 0.5% FBS, and 10^6^ cells were transferred into the inserts and either left untreated or treated with PEVs. RPMI containing 10% FBS was added to lower chamber as chemotactic stimulus. After 24 h, cells that moved in the lower chamber were collected and counted using a hemocytometer and Olympus CK40 microscope (Olympus Corporation, JPN).

### Analysis of apoptosis

Jurkat cells (10^6^ cell/well) were seeded in a 24-well plate and incubated for 24 h with 1 µM A23817 either in the absence, or in the presence of 30 μg/ml PEVs. Appropriate untreated samples were prepared as control. After 24 h, cells were harvested, spun at 700 × *g* for 7 min, resuspended in PBS and counted. Cells (5 × 10^4^) were transferred into a new tube and incubated with annexin V-APC/propidium iodide (PI) staining mix following the manufacturer’s instructions. Samples were analyzed within 30 min using a BD FACSLyric cytometer (BD Biosciences). Apoptosis was expressed as the percentage of cells in Q2 and Q3 quadrants, which represent the cells in late and early apoptosis, respectively. Necrotic cells were quantified as PI-positive cells in the Q1 quadrant.

### Statistical analysis

All the reported figures are representative of at least three different experiments and the quantitative data are reported as mean ± SD. Comparisons between two groups were done using Student *t*-test, whereas multiple comparisons were performed using one-way analysis of variance (ANOVA) with Bonferroni’s post hoc test. *p*-value less than 0.05 was considered statistically significant. Data were analyzed using GraphPad Prism Version 8.0 software.

## Supplementary information

table s1

table s2

supplemental methods

supplemental figures

uncropped blots

## Data Availability

All data generated or analyzed during this study are included in this published article (and its supplementary information files).
