# Supplementary material for: Proteomic and functional profiling of platelet-derived extracellular vesicles released under physiological or tumor-associated conditions
Source: Cell Death Discov. 2022 Nov 26;8:467. doi: 10.1038/s41420-022-01263-3 (PMC9701234; doi:10.1038/s41420-022-01263-3)
Supplement: Supplementary file 3 — supplemental methods [file 41420_2022_1263_MOESM3_ESM.docx]

***Supplementary methods***

***Proteomic analysis***

Purified PEVs were digested with trypsin. 40 µg of protein were reduced in 25 µl of 100 mM NH_4_HCO_3_ with 2.5 μL of 200 mM DTT at 90 °C for 20 min and then alkylated with 10 μL of 200 mM iodoacetamide for 1h at RT in dark conditions. Iodoacetamide excess was removed by the addition of 200 mM DTT. The samples were then overnight digested at 37°C. The digests were dried by Speed Vacuum and then desalted on the Discovery® DSC-18 solid phase extraction (SPE) 96-well Plate (25 mg/well) (Sigma-Aldrich Inc., St. Louis, MO, USA) as reported elsewhere [1].

Trypsin-digested sample proteins were then analyzed with a micro-LC Eksigent Technologies (Eksigent Technologies, Dublin, CA, USA) system coupled with a 5600+ TripleTOF system (Sciex, Concord, ON, Canada) equipped with DuoSpray Ion Source. The stationary phase was a Halo C18 column (0.5 x 100 mm, 2.7 µm; Eksigent Technologies, Dublin, CA, USA). The mobile phase was a mixture of 0.1% (v/v) formic acid in water (A) and 0.1% (v/v) formic acid in acetonitrile (B), eluting at a flowrate of 15.0 µL min−1 at an increasing concentration of solvent B from 2% to 40% in 30 min. For identification purposes the samples were subjected to a data dependent acquisition (DDA): the mass spectrometer analysis was performed using a mass range of 100–1500 Da (TOF scan with an accumulation time of 0.25 s), followed by a MS/MS product ion scan from 200 to 1250 Da (accumulation time of 5.0 ms) with the abundance threshold set at 30 cps (35 candidate ions can be monitored during every cycle). The samples were subjected to label-free quantification through a cyclic data independent analysis (DIA) of the mass spectra, using a 25-Da window: the mass spectrometer was operated such that a 50-ms survey scan (TOF-MS) was performed and subsequent MS/MS experiments were performed on all precursors. These MS/MS experiments were performed in a cyclic manner using an accumulation time of 40 ms per 25-Da swath (36 swaths in total) for a total cycle time of 1.5408 s. The ions were fragmented for each MS/MS experiment in the collision cell using the rolling collision energy. The MS data were acquired with Analyst TF 1.7 (Sciex, Concord, ON, Canada). Every sample was analyzed with one DDA and one DIA acquisitions. The DDA files were searched using Protein Pilot software v. 4.2 (Sciex, Concord, ON, Canada) and Mascot v. 2.4 (Matrix Science Inc., Boston, MA, USA) using trypsin as enzyme, with 2 missed cleavages, a search tolerance of 50 ppm for the peptide mass tolerance, and 0.1 Da for the MS/MS tolerance. The UniProt Swiss-Prot reviewed database containing human proteins (version 01/02/2018, containing 42271 sequence entries), with a false discovery rate fixed at 1%.

The label-free quantification was performed by integrating the extracted ion chromatogram of all the unique ions for a given peptide. The quantification was carried out with PeakView 2.0 and MarkerView 1.2. (Sciex, Concord, ON, Canada). Six peptides per protein and six transitions per peptide were extracted from the SWATH files. Shared peptides were excluded as well as peptides with modifications. Peptides with FDR lower than 1.0% were exported in MarkerView for the t-test (p-value < 0.05 and fold change > 1.3). Bioinformatic analysis was carried out using Ingenuity Pathways Analysis (IPA) software (Qiagen, Redwood City, CA, USA) [2].

**Bibliography**

1. Brandi J, Cheri S, Manfredi M, et al (2020) Exploring the wound healing, anti-inflammatory, anti-pathogenic and proteomic effects of lactic acid bacteria on keratinocytes. Sci Rep 10:11572

2. Manfredi M, Brandi J, Di Carlo C, Vita Vanella V, Barberis E, Marengo E, Patrone M, Cecconi D (2019) Mining cancer biology through bioinformatic analysis of proteomic data. Expert Rev Proteomics 16:733–747
