# Supplementary material for: Proteomic and functional profiling of platelet-derived extracellular vesicles released under physiological or tumor-associated conditions
Source: Cell Death Discov. 2022 Nov 26;8:467. doi: 10.1038/s41420-022-01263-3 (PMC9701234; doi:10.1038/s41420-022-01263-3)
Supplement: Supplementary file 4 — supplemental figures [file 41420_2022_1263_MOESM4_ESM.docx]

***Supplementary figure 1***

***
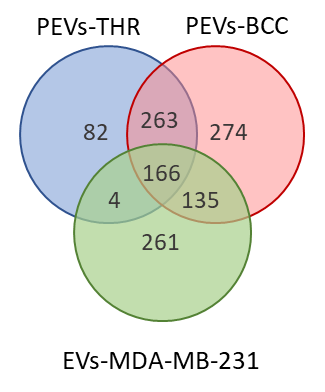
***

**Figure S1**

Venn diagram of proteins identified by LC-MS in PEVs samples and in MDA-MB-231 cell derived-extracellular vesicles (EVs-MDA-MB-231).

***Supplementary figure 2***

***
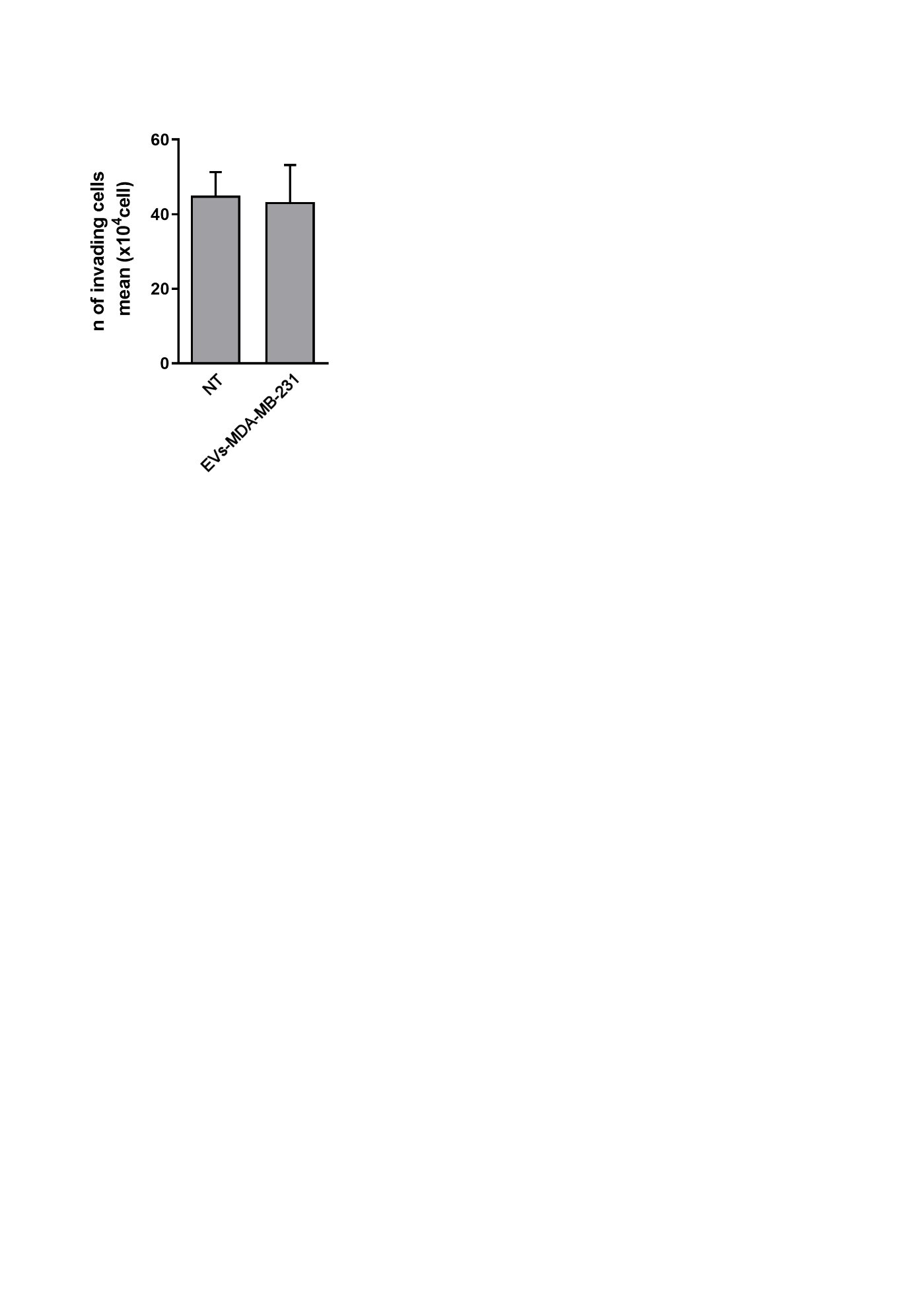
***

**Figure S2**

Cell migration evaluated by trans-well based assay upon treatment of Jurkat cell with EVs-MDA-MB-231. The data are expressed as number of migrated cells and the plots show the mean ± SD of three independent experiments.

***Supplementary figure 3***

***
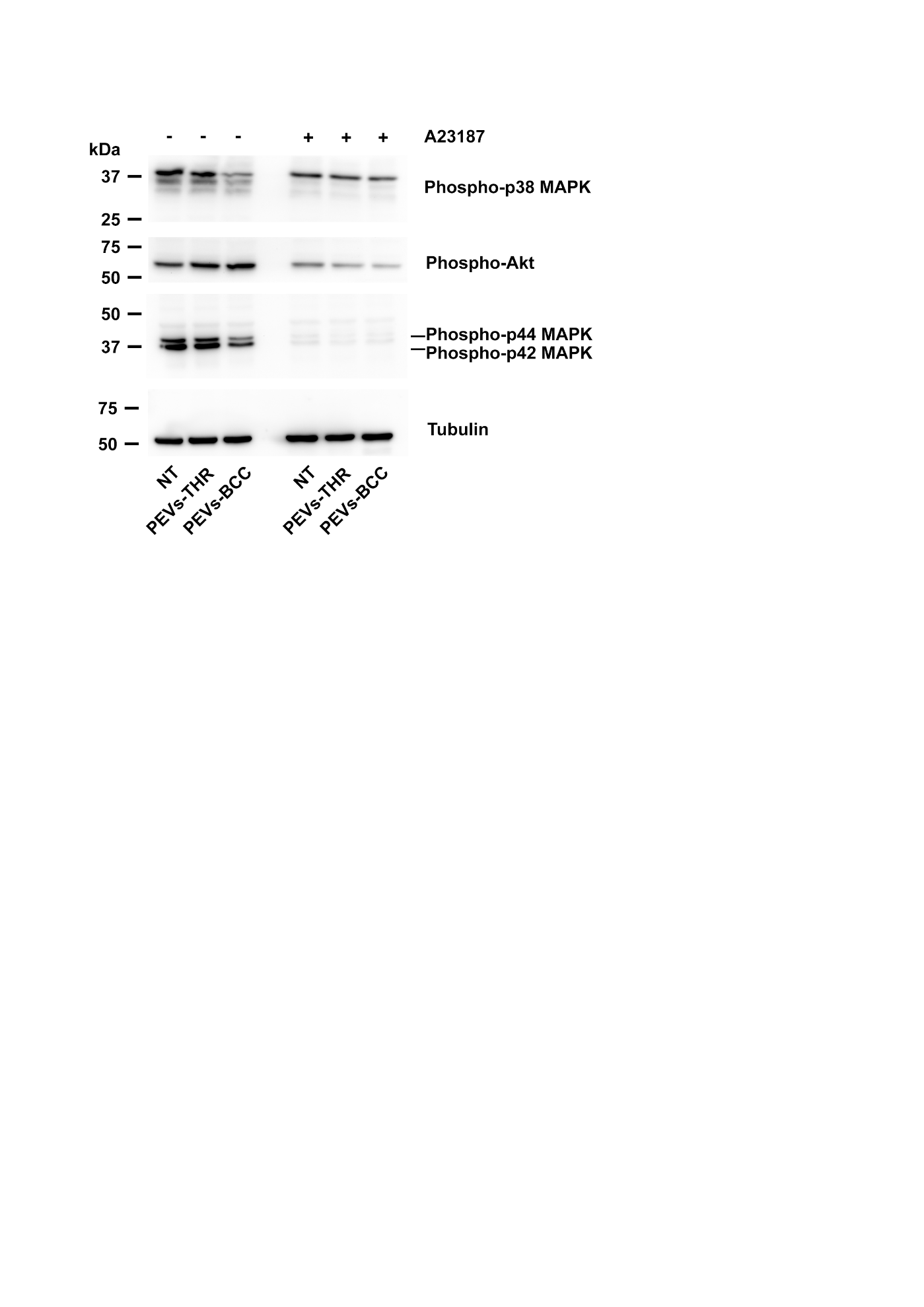
***

**Figure S3**

Representative immunoblotting analysis of the activation of signaling proteins involved in apoptosis. The figure shows the phosphorylation of p38 MAPK, p42/44 MAPK and Akt in Jurkat cells upon incubation with PEVs-THR and PEVs-BCC in the presence or absence of A23187 (indicated as + or – in the figure, respectively). Tubulin staining is for equal loading control.
