# Supplementary figures and images for: Proteomic and functional profiling of platelet-derived extracellular vesicles released under physiological or tumor-associated conditions
Source: Cell Death Discov. 2022 Nov 26;8:467. doi: 10.1038/s41420-022-01263-3 (PMC9701234; doi:10.1038/s41420-022-01263-3)

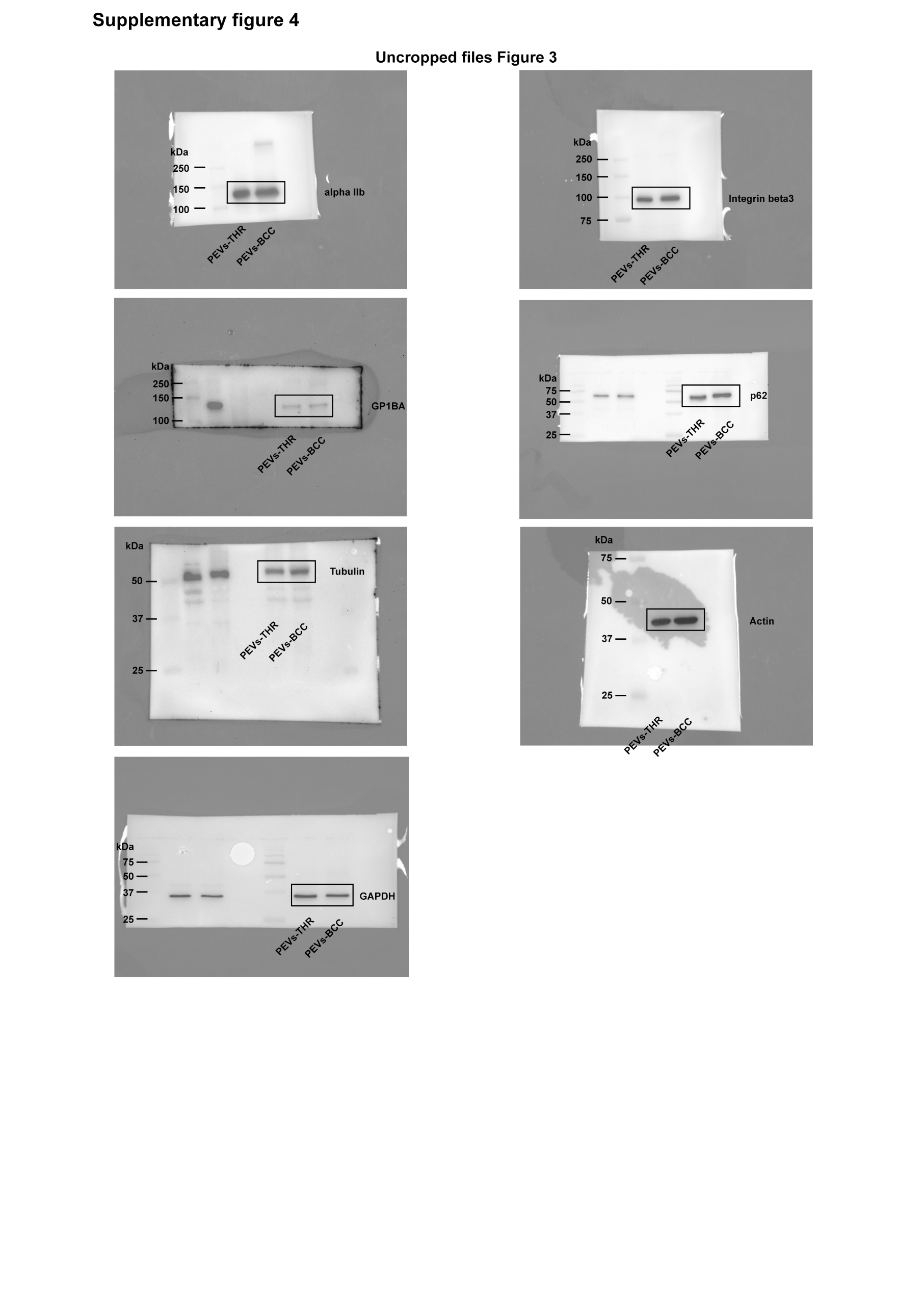


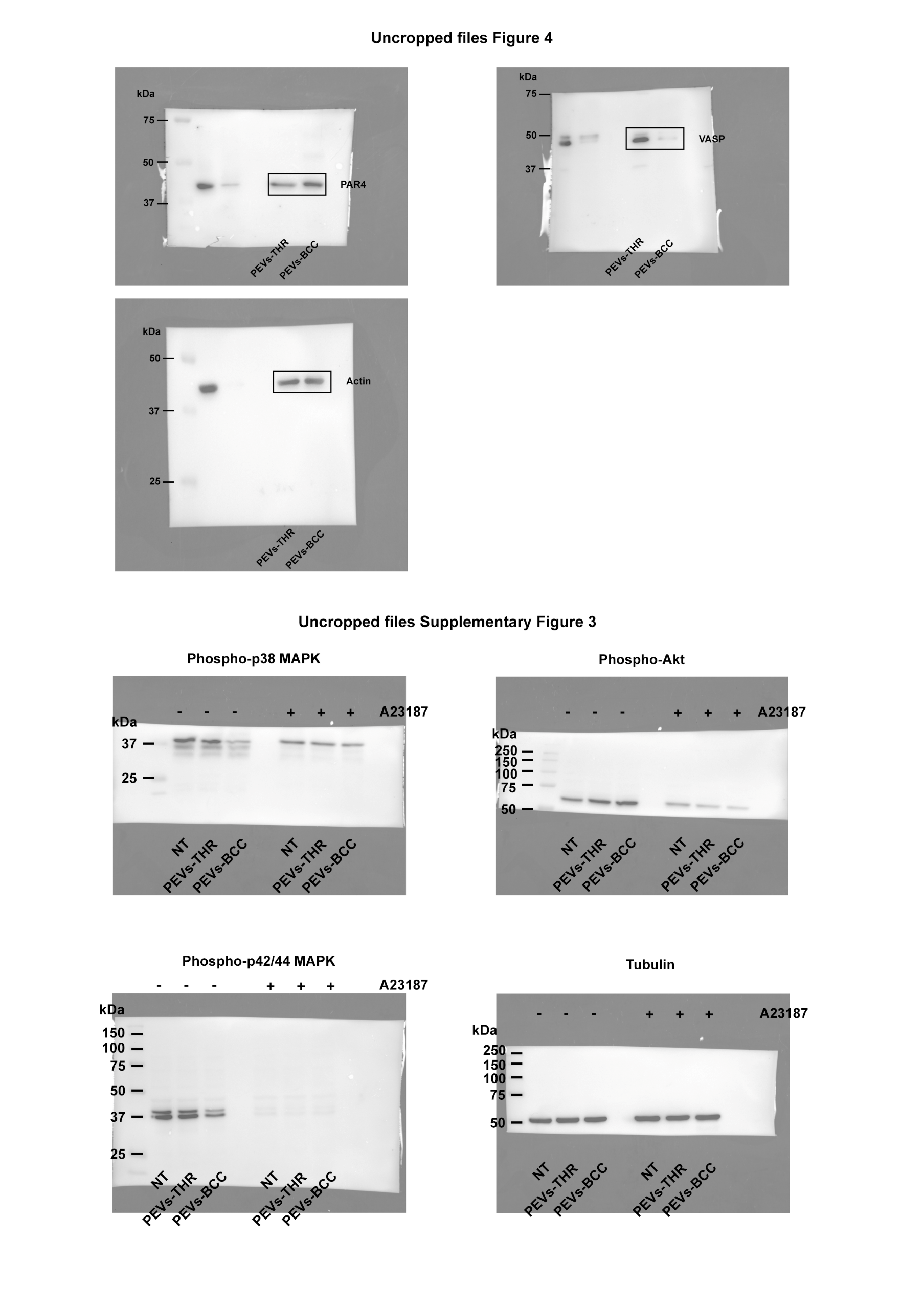

Supplement: Supplementary file 5 — uncropped blots [file 41420_2022_1263_MOESM5_ESM.docx]
